# In-situ anodic precipitation process for highly efficient separation of aluminum alloys

**DOI:** 10.1038/s41467-021-26119-9

**Published:** 2021-10-01

**Authors:** Yu-Ke Zhong, Ya-Lan Liu, Kui Liu, Lin Wang, Lei Mei, John K. Gibson, Jia-Zhuang Chen, Shi-Lin Jiang, Yi-Chuan Liu, Li-Yong Yuan, Zhi-Fang Chai, Wei-Qun Shi

**Affiliations:** 1 Laboratory of Nuclear Energy Chemistry, Institute of High Energy Physics, Chinese Academy of Sciences, 100049 Beijing, China; 2 University of Chinese Academy of Sciences, 100049 Beijing, China; 3 Sino-French Institute of Nuclear Engineering and Technology, Sun Yat-sen University, 519000 Zhuhai, China; 4 Chemical Sciences Division, Lawrence Berkeley National Laboratory (LBNL), Berkeley, CA 94720 USA; 5 Engineering Laboratory of Advanced Energy Materials, Ningbo Institute of Industrial Technology, Chinese Academy of Sciences, 315201 Ningbo, China

**Keywords:** Electrochemistry, Nuclear chemistry, Process chemistry

## Abstract

Electrorefining process has been widely used to separate and purify metals, but it is limited by deposition potential of the metal itself. Here we report in-situ anodic precipitation (IAP), a modified electrorefining process, to purify aluminium from contaminants that are more reactive. During IAP, the target metals that are more cathodic than aluminium are oxidized at the anode and forced to precipitate out in a low oxidation state. This strategy is fundamentally based on different solubilities of target metal chlorides in the NaAlCl_4_ molten salt rather than deposition potential of metals. The results suggest that IAP is able to efficiently and simply separate components of aluminum alloys with fast kinetics and high recovery yields, and it is also a valuable synthetic approach for metal chlorides in low oxidation states.

## Abstract (web summary)

Traditional electrorefining process is limited by deposition potential of the metal itself. Here, the authors explore an in-situ anodic precipitation process based on different solubility of target metal chlorides that can efficiently separate components of aluminum alloys.

## Introduction

Attributes of aluminum and its alloys include light weight, high strength and excellent corrosion resistance, which have led to widespread applications^1^. In the nuclear field, aluminum is a component of fuel (UO_2_/Al, U-Si/Al, U-Mo/Al, and U-Al/Al) and fuel cladding^2^, and is also among the most promising metallic solvents and electrode materials for actinide-lanthanide pyroprocessing separations^3^. However, handling the resulting radioactive aluminum alloys by aqueous reprocessing can be challenging as its use of strong acids for dissolution may be complex and will generate abundant radioactive liquid waste^3^, while by chlorination route use of reactive gases like Cl_2_ or HCl for high-temperature chlorination will corrode equipment and present additional hazards^4^.

Electrochemical technologies are widely used in synthesis^5–7^, metal extraction^8,9^, energy conversion^10–13^, and separations^14,15^. Due to unique features enabled by physical separation of the cathode and anode, an electrochemical technique can be tailored for specific components, often with high efficiency and product purity, and at low cost^16^. In a typical electrochemical separation the cathode potential is controlled to selectively deposit the desired product, while other components remain dissolved in the electrolyte, with the separation efficiency dependent on the difference in reduction potentials of the desired product and the other components^17^. However, the traditional electrochemical separation approaches are often challenging or impractical for Al alloys, especially those containing active metals that are electrochemically oxidized at more negative potentials than oxidation potential of Al^0^/Al^3+^^18,19^. Usually, these active metals will preferentially dissolve at the anode to form metal ions, and co-reduce with Al^3+^ at the cathode to form Al alloys again due to the small difference deposition potential of these active metals, making them difficult to be separated.

Here, we report a different anodic process, in-situ anodic precipitation (IAP), to separate target metals from aluminum alloy components. In IAP, the target metals can be precipitated at the anode immediately after oxidation, by combining with chloride in the NaAlCl_4_ molten salt electrolyte, in contrast to typical electrochemical approaches in which soluble ions or gases like O_2_, Cl_2_, or CO_2_ are generated at the anode. The key to IAP for the separation is thus a difference in metal ion solubilities, rather than a difference in reduction potentials of metals. To evaluate the IAP method, results are reported for separation of aluminum from typical alloy constituents U, Ti, and representative lanthanides. In addition to demonstrating IAP as effective for separation of Al alloys, the results reveal it as a different approach to prepare metal chlorides in low oxidation states.

## Results

### Overview of the IAP process

**Fig. 1:**
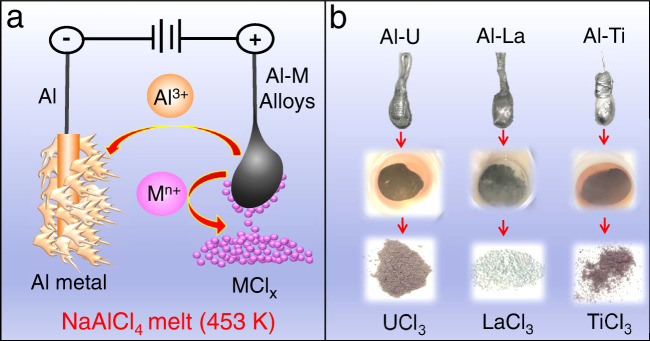
Schematic of the IAP process. **a** Schematic illustration of the IAP process in NaAlCl_4_ molten salt electrolyte. **b** Various alloys and corresponding metal chlorides precipitated in the bottom of crucible by electrolysis with different anodes and final metal chloride products obtained.

Fig. 1a is a schematic diagram of the IAP process for separating Al from a metal M in binary alloy Al-M, and Fig. 1b presents the images obtained by electrolysis with different anodes. The anode is the alloy and the cathode is a conductive metal such as pure Al. In operation, alloy constituents M and Al are oxidized to M^n+^ and Al^3+^. While Al^3+^ is soluble in the electrolyte^20^, as AlCl_4_^−^ or Al_2_Cl_7_^2−^, insoluble M^n+^ formed at the anode rapidly combines with chloride anions to form an in-situ precipitate of solid MCl_x_ very close to the anode surface. Co-generation of soluble Al species at the anode induces continuous flaking and removal of the precipitate from the anode surface, which maintains an electrochemically active interface. At the cathode, dissolved Al^3+^ is reduced to aluminum metal and co-deposited with NaCl. Advantages of NaAlCl_4_ molten salt as the electrolyte include good ionic conductivity, a low melting point of 426 K with resultant low operating temperature, and low density and viscosity^20,21^. The IAP separation approach is enabled by low solubility of actinide, lanthanide, and transition metal chlorides in the electrolyte at the operating temperature, for neutral or alkaline conditions with AlCl_3_ content of 50 mol% or less^22,23^. The low viscosity and density of the melt facilitates detachment of the precipitate from the anode, with product accumulation beneath the anode at the bottom of the electrochemical cell enabling separation by simply decanting the electrolyte.

### Separation of U and Al

**Fig. 2:**
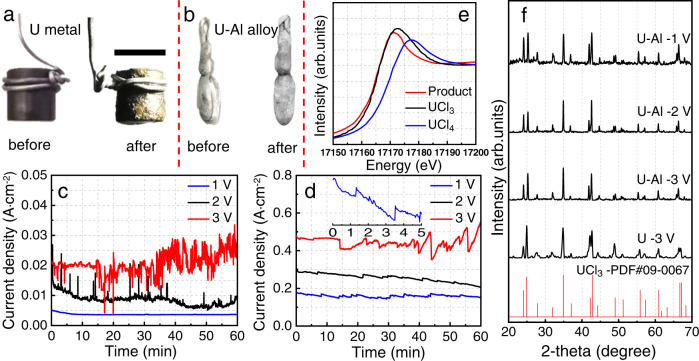
Anodic processes of U and Al-U alloy. Images of **a** U metal anode and **b** Al-U alloy anode before and after electrolysis. Scale bar = 1 cm. Current versus time plots for three electrode potential using **c** U anode and **d** Al-U alloy anode in NaAlCl_4_ melt at 453 K. **e** U L_3_-edge XANES spectra of standards UCl_4_ and UCl_3_, and the U anode product of electrolysis at 3 V vs. Al (quenched suddenly to the solid state at room temperature without any other treatment). **f** XRD patterns of anode products after electrolysis of pure U at 3 V and the Al-U alloy at 1 V, 2 V and 3 V vs. Al; and standard pattern for UCl_3_.

**Fig. 3:**
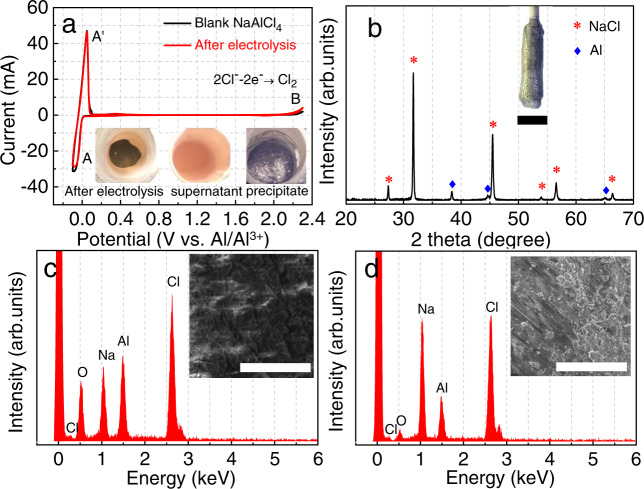
Analyses of electrolyte and cathode after IAP. **a** CV curves of NaAlCl_4_ melt before (black line) and after electrolysis (red line); insets are photos looking down into the anode crucible after electrolysis, and of the separated supernatant and precipitate. **b** XRD pattern of the cathode product after electrolysis, with a photo of the cathode. Typical EDS analyses of **c** electrolyte and **d** cathode product after electrolysis; insets are the corresponding SEM images. The scale of **b** is 1 cm and **c**, **d** is 100 µm.

Pure U metal and Al-U alloy anodes were both employed to demonstrate general performance of IAP. These anodes before and after electrolysis are shown in Fig. 2a, b, where it is apparent that both surfaces were highly corroded by electrolysis, with the pure U anode particularly damaged and coarse. Fig. 2c, d show the current versus time curves for U and Al-U anodes at three potentials. No significant current for the U anode is observed for an applied potential of 1 V vs. Al, presumably because adhesion of initial oxidation products prevents further reaction. As the U anode potential is increased to 2 V and then 3 V vs. Al, the current density increases, which suggests that anode passivation is overcome, with a result that kinetics are highly dependent on anode polarization. Passivation of the anode is evidently rate-determining, and a high electrode potential greatly accelerates the IAP process. The inset of Fig. 2d shows marked current oscillations with electrolysis time, possibly with some periodicity. The IAP oscillations presumably reflect precipitation and elimination of UCl_3_ from the anode (Supplementary Fig. 1). X-ray diffraction (XRD) analysis such as in Fig. 2f reveals crystalline product UCl_3_, with no apparent side reactions even for an anodic oxidation potential above that for evolution of Cl_2_ (2.30 V vs. Al shown in Fig. 3a). Extended X-ray absorption fine structure (EXAFS) characterization (Fig. 2e and Supplementary Fig. 2, Supplementary Table 1) of the IAP anodic precipitate after electrolysis at 3 V provided the U oxidation state and local coordination environment^24,25^ in good agreement with XRD (Fig. 2f). Given that U^3+^ is typically oxidized to U^4+^ at such a high potential, this result suggests U^3+^ is trapped in the precipitate, preventing its further oxidation.

Current vs. time curves for the Al-U alloy anode exhibit the same general behavior as for the U anode, but with greater current density of up to 0.5 A/cm^2^ at 3 V vs. Al (Fig. 2d). The higher current for Al-U suggests that formation of soluble ions like AlCl_4_^−^ promotes detachment of precipitates which maintains an active electrode surface. For the U and Al-U electrodes at 3 V vs. Al, the current density generally increases with the time of the electrolysis (Fig. 2c, d), possibly due to an effect of active electrode surface increase. As U is oxidized, the anode surface gets more “structured”, uneven, and rough (Supplementary Fig. 1b), which leads to an increase of anodic active surface. On the other hand, smilar senario on the cathode occurs as dendritic aluminum deposited at the same time, which increases the cathodic active surface, then the current will increase correspondingly. XRD analysis of the UCl_3_ precipitate from the Al-U anode indicates highly crystalline material. Cyclic voltammograms (CV) of the Al-U anode product in LiCl-KCl reveals no features due to metals other than U (Supplementary Fig. 3), indicating a purity suitable for use in nuclear fuel cycle^26,27^. The recovery yield of U is 94.6% (Supplementary Eq. 2) as determined from the electrode mass before and after electrolysis. It is worthnoting that the results reported above were achieved by using U-Al alloy anode prepared by melting. On the other hand, we studied the IAP process using an alternative Al-U alloy anode prepared by electrolysis, in which the alloy cann’t be compeletely separated possibly due to its poor mechanical properties (Supplementary Fig. 4), demonstrating the advantages of utilization of alloys prepared by the high temperature processing.

The inset in Fig. 3a shows the electrolyte and precipitate after electrolysis. The clear supernatant electrolyte and dark anode precipitate at the bottom of the crucible are clearly distinct from one another, and are easily separated by simply decanting the electrolyte. The separated electrolyte was analyzed by CV, Scanning electron microscopy-energy-dispersive X-ray spectroscopy (SEM-EDS), and ICP-MS. CV curves of NaAlCl_4_ before and after electrolysis are essentially coincident, as in Fig. 3a. The absence of uranium electrochemical response after IAP in the CV potential window between aluminum electrodeposition at 0 V (peak A) and chlorine evolution ~2.3 V (peak B) indicates low solubility of UCl_3_ in NaAlCl_4_ at 453 K. Consistent with the CV results, U was not detected in the melt by SEM-EDS analysis (Fig. 3c). Furthermore, solubility product constant (*K*sp) of UCl_3_ was determined to be 1.20 × 10^−16^ by ICP-MS, as shown in the Table 1, which once again indicates the concentration of U in the melt is very low. Therefore, the reaction of uranium at the anode can be expressed as Eqs. 1 and 3.

**Table 1:** *K*sp and recovery yields analyses of several different anodes for IAP in NaAlCl_4_ molten salt electrolyte at 453 K.

Anode	Product	*K*sp	Yield (%)
U/U-Al	UCl_3_	1.20 × 10^−16^	94.6
La/La-Al	LaCl_3_	1.15 × 10^−16^	97.0
Sm	SmCl_2_	3.76 × 10^−9^	88.7
Eu	EuCl_2_	2.47 × 10^−9^	90.4
Yb	YbCl_2_	1.83 × 10^−9^	94.1
Ti/Ti-Al	TiCl_3_	5.22 × 10^−14^	80.5

Generally, electrodeposition of Al (Eq. 2) was claimed to be a typically cathodic reaction in AlCl_3_-based melt^20^. However, the XRD pattern (Fig. 3b) of the cathode here indicates it is a mixture of NaCl and Al, in good agreement with SEM-EDS result as shown in Fig. 3d, which is due to the low solubility of NaCl in the melt and its slow dissolution kinetics, making it precipitated in-situ at the cathode, as described in Eq. 4. As a result, the growth of cathode dendrite is much inhibited. Related evidence is clearly shown from the rod-shaped cathode after electrolysis (inset of Fig. 3b), which becomes flake-shaped after further washing (inset of Supplementary Fig. 5b). Therefore, the total process of IAP based on the reactions between anode and electrolyte to produce anode precipitates and cathode products can be depicted in Eqs. 1–5 as follows:

1 $$(+)\,{{{{{{\rm{U}}}}}}}_{({{{{{\rm{anode}}}}}})}\to {{{{{{\rm{U}}}}}}}^{3+}+3\,{{{{{{\rm{e}}}}}}}^{-}$$

2 $$(-)\,{{{{{{\rm{NaAlCl}}}}}}}_{4}+3\,{{{{{{\rm{e}}}}}}}^{-}\to {{{{{{\rm{Al}}}}}}}_{({{{{{\rm{cathode}}}}}})}+4\,{{{{{{\rm{Cl}}}}}}}^{-}+{{{{{{\rm{Na}}}}}}}^{+}$$

3 $${{{{{{\rm{U}}}}}}}^{3+}+3\,{{{{{{\rm{Cl}}}}}}}^{-}\to {{{{{{\rm{UCl}}}}}}}_{3({{{{{\rm{ppte}}}}}})}$$

4 $${{{{{{\rm{Na}}}}}}}^{+}+{{{{{{\rm{Cl}}}}}}}^{-}\to {{{{{{\rm{NaCl}}}}}}}_{({{{{{\rm{ppte}}}}}})}$$

5 $${{{{{{\rm{U}}}}}}}_{({{{{{\rm{anode}}}}}})}+{{{{{{\rm{NaAlCl}}}}}}}_{4}\to {{{{{{\rm{UCl}}}}}}}_{3({{{{{\rm{ppte}}}}}})}+{{{{{{\rm{Al}}}}}}}_{({{{{{\rm{cathode}}}}}})}+{{{{{{\rm{NaCl}}}}}}}_{({{{{{\rm{ppte}}}}}})}$$

The recovery yield of aluminum is up to 99.4%. In addition, XRD and SEM-EDS analyses of the cathode product after washing (Supplementary Fig. 5) confirm its aluminum metal nature, which once again proves that uranium will not be deposited on the cathode. Chlorine and oxygen impurities may respectively result from electrolyte adhesion to the surface and reaction with air. Further, uranium is not detected from the cathode product by ICP-MS. The absence of uranium in both the electrolyte and cathode after IAP demonstrates effective separation of U from the Al-U alloy. However, U is not completely recovered at the anode (94.6%, see Table 1), most likely due to the adhesion of its precipitates on the cathode rather than chemical deposition. This phenomenon is also observed in the IAP of divalent rare earth (Supplementary Fig. 8).

### Separation of lanthanides and titanium from Al

**Fig. 4:**
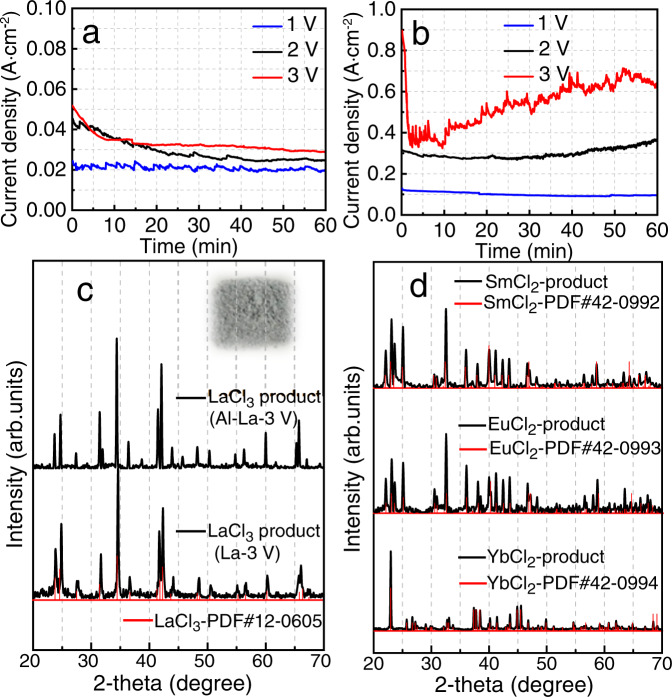
Anodic processes of lanthanides. Current vs. time plots at three electrode potentials in NaAlCl_4_ melt at 453 K using as the anode **a** La and **b** Al-La alloy. **c** XRD patterns of IAP anode precipitates for La and Al–La anodes; the inset is a photo of the LaCl_3_ product. **d** XRD patterns of precipitates from Sm, Eu, and Yb anodes.

To assess its wider applicability, the IAP approach is extended to Al-lanthanide and Al-titanium alloys. Results for La, a representative lanthanide, are in Supplementary Fig. 6, where it is apparent that surface material is removed from both the La and Al-La alloy anodes during electrolysis. Figure 4a, b show current versus time curves for these anodes at three potentials; addition of Al to La greatly enhances the dissolution rate, as seen above when comparing U and Al-U. The grayish anode product shown in the inset of Fig. 4c is identified by XRD as crystalline LaCl_3_. Also studied as IAP anodes were pure Sm, Eu, and Yb, and both pure Ti and an Al-Ti alloy (Supplementary Figs. 7–9). All four of these metals are precipitated in low oxidation states in the anode products, specifically SmCl_2_, EuCl_2_, YbCl_2_, and TiCl_3_ as shown in Fig. 4d and Supplementary Fig. 9d. These oxidation states are obtained at an anodic oxidation potential of 3.0 V vs. Al, which is above the limit for chlorine evolution. Retention of low oxidation states is further demonstration that the composition of the anode product is largely independent of the applied potential, which contrasts with usual electrochemical behavior. The much slower oxidation kinetics for anodes of Ti and Al–Ti alloy (Supplementary Fig. 9a, b), vs. La and Al–La, presumably reflects some sort of passivation of Ti. Formation of trivalent Ti as TiCl_3_ as the anode precipitate product is particularly remarkable. In addition, high-temperature vaporization to separate TiCl_3_ from NaAlCl_4_ transforms trivalent Ti to other oxidation states^28,29^, which is not obeserved in the divalent lanthanide chlorides. A separate later effort will further assess IAP for synthesis of compounds containing Ti (III) and other low oxidation state metals (Supplementary Fig. 10).

The *K*sp and recovery yields of several different anodes for IAP are listed in Table 1. The recovery yields of trivalent chlorides are significantly higher than that of divalent ones, suggesting very low solubility of trivalent chlorides in this electrolyte, which is well in agreement with the evolution trend of *K*sp. For the divalent chlorides, the recovery yield of YbCl_2_ is higher than that of SmCl_2_ and EuCl_2_, which is correlated with the lower solubility of divalent Yb in the electrolyte and is also consistent with *K*sp and EDS results (Supplementary Fig. 8). In addition, YbCl_2_ often falls off from the anode in big flakes, making it even easier to be completely separated and recovered from the electrolyte.

## Discussion

Herein, the IAP process has been demonstrated in NaAlCl_4_ molten salt electrolyte, using several metals and their alloys with Al. Especially, efficiently direct separation of U–Al alloys has been achieved in a one-step manner with high-purity UCl_3_ precipitate at the anode and pure Al deposited at the cathode under mild conditions with high recovery yield. As the IAP method employs different solubilities of anode products, rather than different reduction potentials for deposition, it is not limited by an electrochemical window or cathode potential. In addition, increasing the anodic potential and/or the aluminum content in the alloy provides rapid oxidation/precipitation kinetics. Furthermore, the simplicity of IAP indicates it as a practical approach for the separation of active metals in aluminum alloys and, in light of the results presented in this study, shows promise for extension to the production of high-purity and low oxidation state compounds at low temperatures. It is inspiring that the so-called IAP approach we propose could be possibly extended to other electrolytes, not only based on AlCl_3_, but also other Lewis acid electrolyte and room temperature electrolytes^21,30–32^. More in-depth studies are needed to optimize the method; the initial results reported here suggest broad applicability for separations and material preparation.

## Methods

### Electrolytic apparatus and procedures

The photograph of experimental setup are shown in Supplementary Fig. 11. An alumina crucible (inner diameter: 52 mm) containing ~150 g NaAlCl_4_ electrolyte was fixed in a specified programmable electric furnace. Metals or aluminum alloys were used as the working electrode (WE). An aluminum rod (Φ = 3 mm, 99.999%) was the counter electrode (CE), and the reference electrode (RE) was an aluminum wire (Φ = 1 mm, 99.999%) inserted in a pyrex glass tube (Φ = 6 mm) filled with NaAlCl_4_ melt (Supplementary Fig. 11c)^33^. Electrolysis experiments were carried out using an Autolab PGSTAT 302N in a potentiostatic mode, keeping a potential of 1 V, 2 V or 3 V vs. Al, respectively. All operations were performed in a glove box with moisture and oxygen maintained below 3.0 ppm. The temperature of the melt was measured with a K-type thermocouple (±2 K), with the electrolysis temperature maintained at 453 ± 5 K. The surface areas of the electrodes were calculated by measuring the depth of the wetting electrode immersed in NaAlCl_4_ melt after each experiment. At the end of the electrolysis experiment, the setup was left to set at 453 K for 8 h and the upper layer of electrolyte was separated by decanting to obtain the bottom precipitates. Some collected U anode precipitates were quenched for EXAFS analysis. Then, they were distilled at 773 K for 8 h to remove the NaAlCl_4_ molten salt electrolyte adhering to its surface for XRD analyses. However, for the precipitates obtained from Al–Mo, Al–Ti anodes, they were vacuum distilled at 573 K for 1 h because of their poor stability at high temperature^28^.

### Preparation of electrolyte and electrodes

NaAlCl_4_ molten salt electrolyte was prepared using anhydrous sodium chloride (>99.9%) and aluminum chloride (>99.5%)^23^. First, aluminum chloride and sodium chloride mixture (1:1) was put into an alumina crucible, heated to 473 K with a heating rate of 10 K/min and kept at this temperature for 10 h. Then, the melt was naturally cooled to 453 K, and two aluminum rods (Φ = 3 mm, 99.999%) were used as cathode and anode, respectively. Colorless and transparent electrolyte can be obtained after 3 days of electrolysis under the voltage of 1 V vs. Al. The uranium electrode was a cylindrical U metal ingot (99.5%, diameter Φ = 1.0 cm; length = 1.0 cm) wrapped with aluminum wire. The other pure metal anodes (La, Sm, Eu, Yb, and Ti with 99.5% purity) were prepared similarly. The Al–U alloys were ~5 mm in diameter which were prepared by melting uranium (60 wt%) and aluminum (99.999%) in a vacuum furnace; the Al–La, Al–Mo, and Al–Ti alloys were prepared similarly. Prior to the experiments, all anodes were polished with 1200 mesh silicon carbide sandpaper and cleaned with alcohol. The detailed conditions for CV testing are described in Supplementary methods.

### Characterizations

X-ray absorption near edge structure and EXAFS spectra at the U L_3_-edge were collected at beamline 1W1B of the Beijing Synchrotron Radiation Facility using transmission mode^34^. XRD (Bruker, D8 Advance) was used for phase analysis. SEM (Hitachi S-4800)-EDS (GENESIS 2000) were used for surface morphology and composition analysis.

## Supplementary information

Supplementary Information

## Data Availability

The authors declare that all data supporting the findings of this study are either provided in the article and its Supplementary Information or available from the corresponding author upon request.
