# Supplementary material for: In-situ anodic precipitation process for highly efficient separation of aluminum alloys
Source: Nat Commun. 2021 Oct 1;12:5777. doi: 10.1038/s41467-021-26119-9 (PMC8486879; doi:10.1038/s41467-021-26119-9)
Supplement: Supplementary file 1 — Supplementary Information [file 41467_2021_26119_MOESM1_ESM.pdf]

Supplementary Information

In-situ Anodic Precipitation Process for Highly Efficient Separation of Aluminum Alloys

Zhong et al.

Supplementary Methods

CV tests. For CV tests in $NaAlCl_4$ molten salt electrolyte at 453 K, a tungsten wire (Φ=1 mm, 99.999%), an aluminum rod (Φ=3 mm, 99.999%) and an aluminum wire (Φ=1 mm, 99.999%) were used as the WE, CE and RE, respectively (Supplementary Fig. 12a). For CV tests in LiCl-KCl melt at 723 K, a tungsten wire (Φ=1 mm, 99.999%), a platinum wire (Φ=1 mm, 99.99%) and a graphite rod (Φ= 6 mm, spectral purity) were used as the WE, RE and CE, respectively (Supplementary Fig. 12b).

Calculations of recovery yield. Recovery yield of anode products was calculated from the follows (Supplementary Eq. 1-8):

$$M_{\text{Recovery yield}} = \frac{m_{\text{precipitate}}}{(m_{\text{anode}} - m_{\text{anode after electrolysis}}) * \frac{m_{\text{metal}}}{m_{\text{alloy}}} * \frac{M_{\text{metal}} + n * M_{\text{Cl}}}{M_{\text{metal}}}} * 100\% \quad (1)$$

Here, m is measured mass, M is the molar mass, and n is the oxidation state of the metal in the precipitate.

$$U_{\text{Recovery yield}} = \frac{0.92\text{ g}}{(9.32\text{ g} - 8.20\text{ g}) * 60\% * \frac{238.0 + 3 * 35.45}{238.0}} * 100\% = 94.6\% \quad (2)$$

$$La_{\text{Recovery yield}} = \frac{3.24\text{ g}}{(14.08\text{ g}-12.19\text{ g}) * \frac{138.9 + 3 * 35.45}{138.9}} * 100\% = 97.0\% \quad (3)$$

$$Sm_{\text{Recovery yield}} = \frac{2.36\text{ g}}{(7.66\text{ g} - 5.85\text{ g}) * \frac{150.4 + 2 * 35.45}{150.4}} * 100\% = 88.7\% \quad (4)$$

$$Eu_{\text{Recovery yield}} = \frac{1.51\text{ g}}{(5.23\text{ g} - 4.09\text{ g}) * \frac{152.0 + 2 * 35.45}{152.0}} * 100\% = 90.4\% \quad (5)$$

$$Yb_{\text{Recovery yield}} = \frac{1.74\text{ g}}{(6.82\text{ g}- 5.51\text{ g}) * \frac{173.0 + 2 * 35.45}{173.0}} * 100\% = 94.1\% \quad (6)$$

$$Ti_{Recovery\ yield} = \frac{1.64\ g * 48.4\%}{(6.10\ g - 5.59\ g) * 60\% * \frac{47.87 + 3 * 35.45}{47.87}} * 100\% = 80.5\% \quad (7)$$

The content of Ti in the precipitate is 48.4%.

For separation of U-Al alloy: $Al_{Recovery\ yield}$ =

$$\frac{m_{cathode}}{(m_{anode} - m_{anode\ after\ electrolysis}) * 40\ \% + (m_{anode} - m_{anode\ after\ electrolysis}) * 60\ \% * \frac{M_{Al}}{M_U}} * 100\%$$

$$= \frac{0.293\ g}{(13.13\ g - 12.50\ g) * 40\ \% + (13.13\ g - 12.50\ g) * 60\ \% * \frac{26.98}{238.0}} * 100\% = 99.4\% \quad (8)$$

Calculations of K_{sp}. K_{sp} of the anode products in $NaAlCl_4$ melt was calculated from the follows (Supplementary Eq. 9-15):

$$K_{sp}(MCl_n) = [M] * [Cl^-]^n \quad (9)$$

$$K_{sp}(UCl_3) = 3.99*10^{-6}*[10^{-3.487}]^3 = 1.20 * 10^{-16} \quad (10)$$

$$K_{sp}(LaCl_3) = 3.84*10^{-6}*[10^{-3.487}]^3 = 1.15 * 10^{-16} \quad (11)$$

$$K_{sp}(SmCl_2) = 3.55*10^{-2}*[10^{-3.487}]^2 = 3.76 * 10^{-9} \quad (12)$$

$$K_{sp}(EuCl_2) = 2.33*10^{-2}*[10^{-3.487}]^2 = 2.47 * 10^{-9} \quad (13)$$

$$K_{sp}(YbCl_2) = 1.73*10^{-2}*[10^{-3.487}]^2 = 1.83 * 10^{-9} \quad (14)$$

$$K_{sp}(TiCl_3) = 1.74*10^{-3}*[10^{-3.487}]^3 = 5.22 * 10^{-14} \quad (15)$$

Here, $[Cl^-]$ was calculated be $10^{-3.487}$ according to the reference[1].

Supplementary Figures

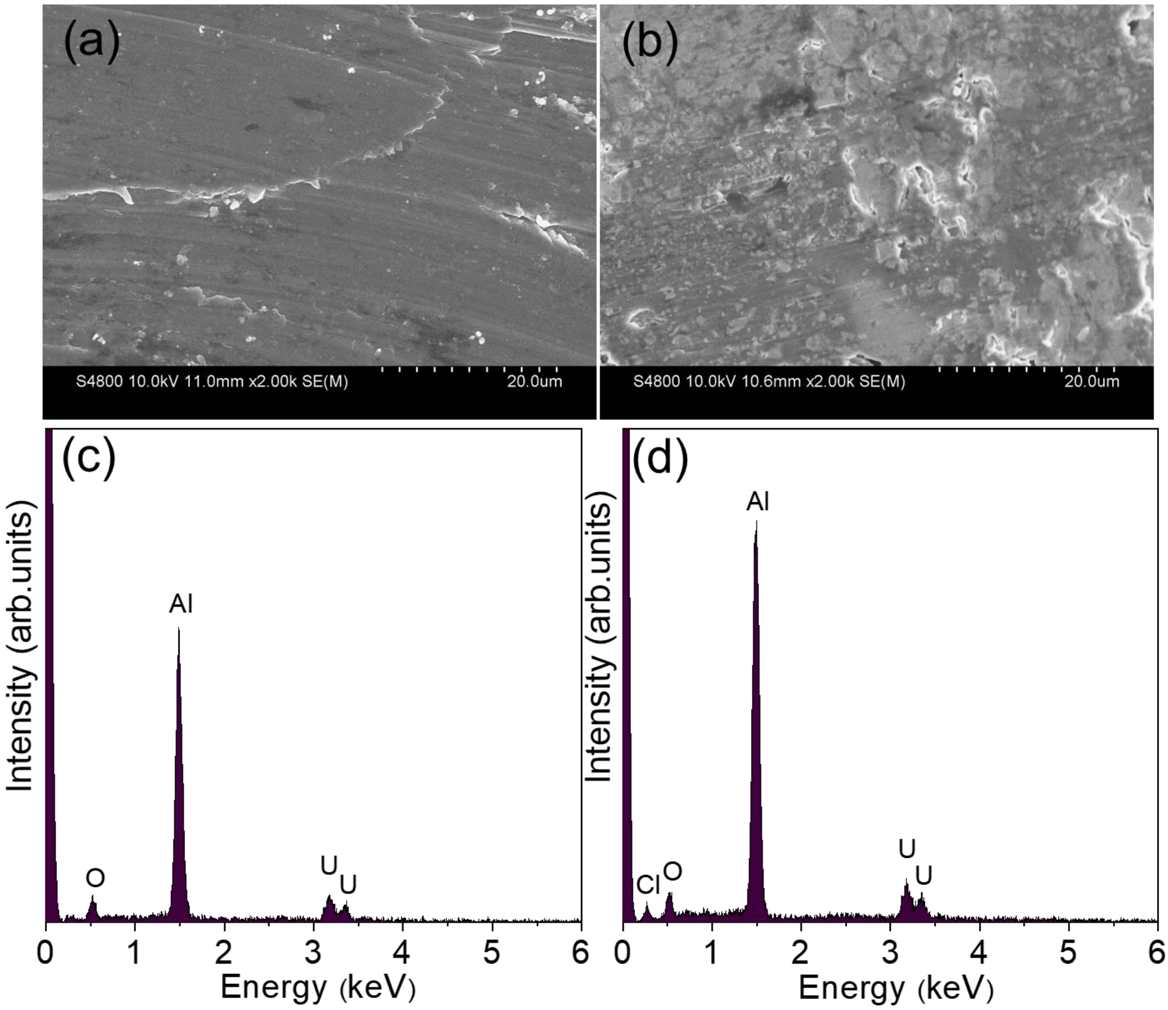

Supplementary Figure 1. Cross-sectional analysis of the anode. Cross sectional SEM images of U-Al alloy anode (a) before and (b) after electrolysis at 3.0 V. Typical EDS analyses of U-Al alloy anode (c) before and (d) after electrolysis at 3.0 V.

Electrode surface modification was assessed from cross sectional SEM images of the U-Al alloy anode before and after electrolysis (Supplementary Fig. 1a and 1b). No obvious morphology changes are apparent, which may reflect dissolution of aluminum resulting in continuous shedding of precipitates and exposure of fresh surface. The chlorine content in the alloy apparent in Supplementary Fig. 2d may be due to adhesion of electrolyte.

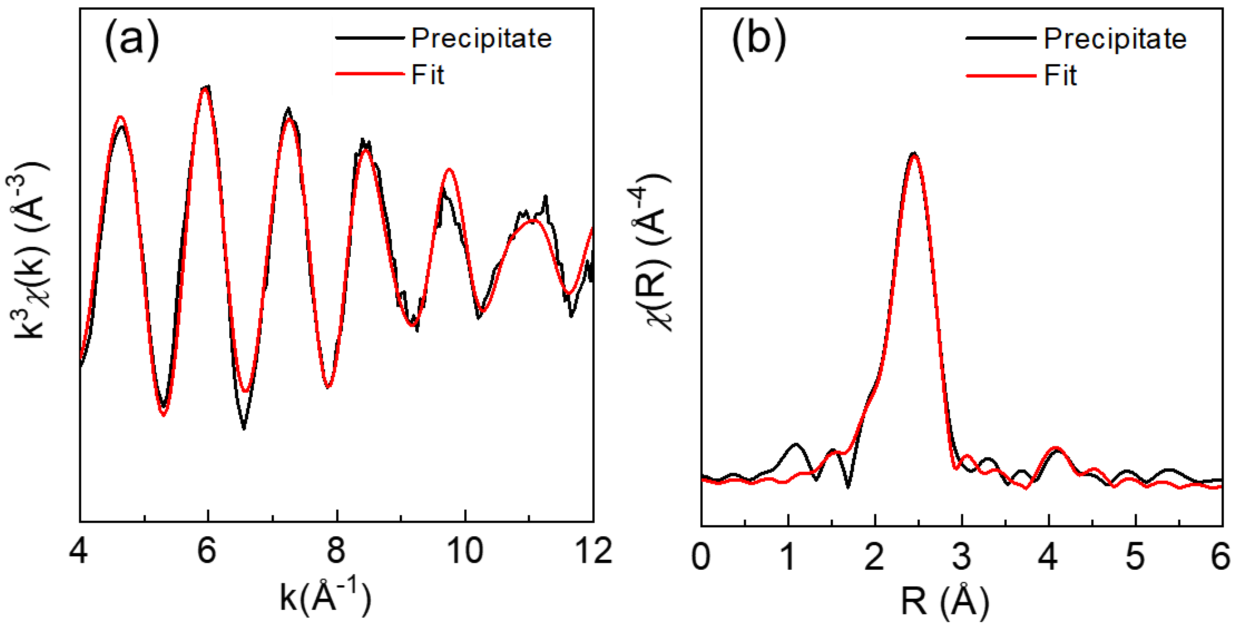

Supplementary Figure 2. The EXAFS analysis of the U anode precipitate. (a) UL_3 edge k3-weighted EXAFS spectra (black line) and best theoretical fits (red line) for IAP precipitate. (b) Corresponding non-phase-shift corrected Fourier transform.

The collected U anode precipitate quenched suddenly (at room temperature) to the solid state for EXAFS analysis to reveal its original local structure. Supplementary Fig. 2 shows U L_3 edge k^3-weighted spectra and their Fourier transforms, with the corresponding fitting results in Supplementary Table I. The results show that the product is UCl_3 with coordination number 6 and bond length 2.92 Å, in good agreement with literature values[2].

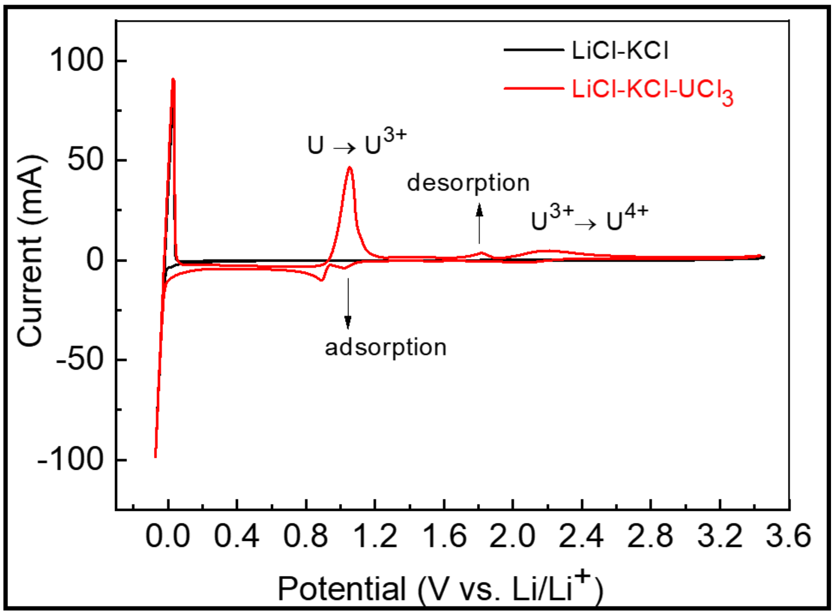

Supplementary Figure 3. Electrochemical analysis of UCl_3 Precipitation. CV curves of UCl_3 (obtained from IAP of U anode) in molten LiCl-KCl at 723 K.

Uranium trichloride is important in pyroprocessing of electrorefining[3]. In order to maintain a stable electrolytic voltage, excess UCl_3 (about 10 wt.%) is added to the electrolyte for electrorefining[4]. To assess the quality of the UCl_3 obtained here, CV was performed in LiCl-KCl melt. As shown in Supplementary Fig. 3, only the uranium redox peak is apparent in the potential window of the melt, indicating high quality UCl_3 suitable for electrorefining[5].

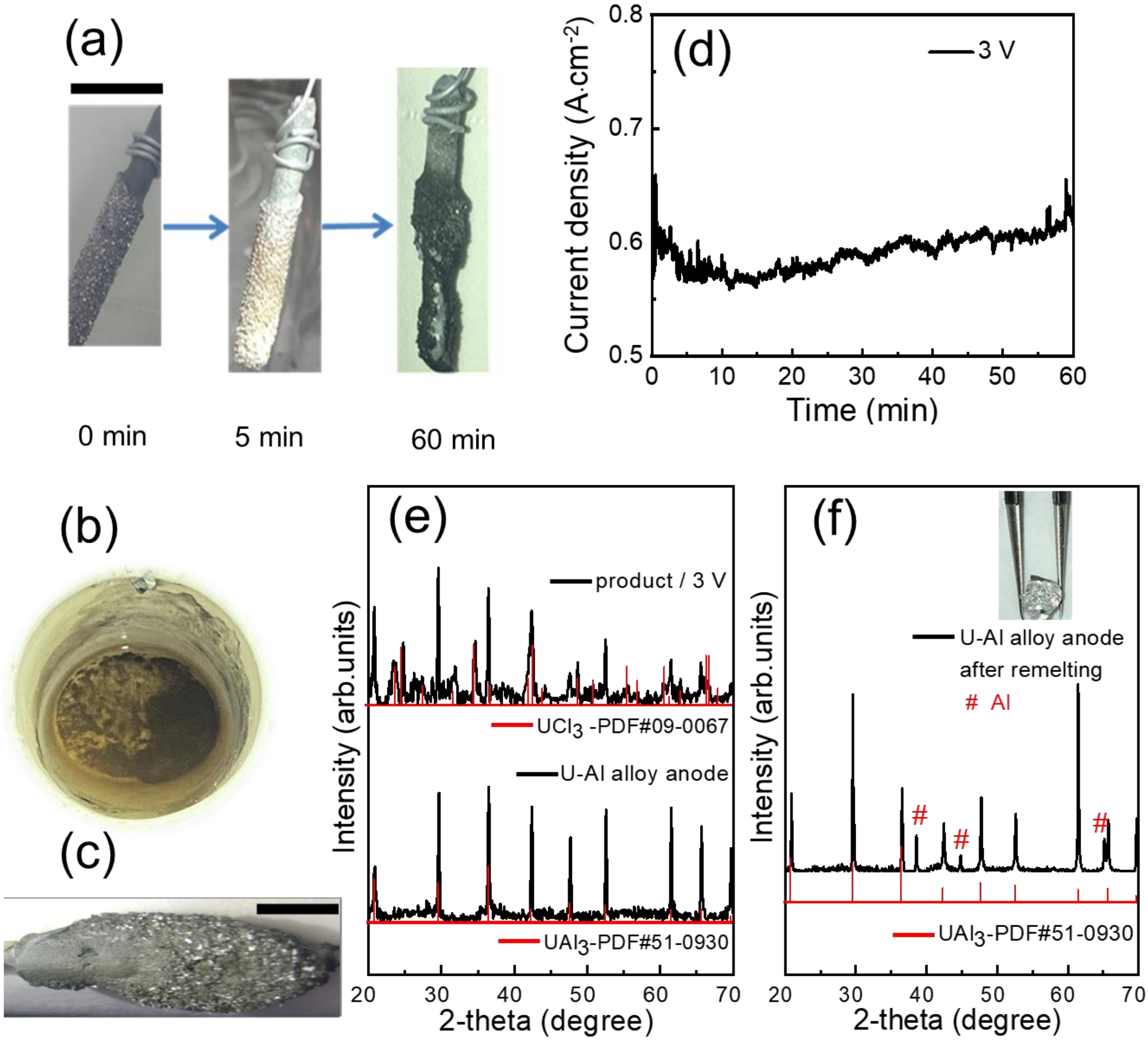

Supplementary Figure 4. IAP process of U-Al alloy prepared by electrolysis. (a) Images of electrolytically-prepared U-Al alloy anode after IAP electrolysis for different times. (b) Photo down into the anode tank after electrolysis. (c) Photo of the cathode after electrolysis. (d) Current versus time plots for the U-Al alloy anode in $NaAlCl_4$ molten salt electrolyte at 453 K. (e) XRD patterns of the U-Al alloy and anode precipitate product. (f) XRD patterns of the U-Al alloy after re-melting (the mass fraction of uranium is 60 %); insets are the cross section of the alloy. Scale bar = 1cm.

In addition to the alloys prepared by melting, U-Al alloys prepared electrolytically[6, 7] were tested as anodes, with results summarized in Supplementary Fig. 4. It is apparent in Supplementary Fig. 4a that metal particles have flaked off the anode surface. The anode

precipitate and electrolyte supernatant after electrolysis are shown in Supplementary Fig. 4b; the precipitate is easily separated from the electrolyte by decanting. The cathode dendritic aluminum product is shown in Supplementary Fig. 4c. In Supplementary Fig. 4d is the current versus time electrolysis curve for an electrode potential of 3 V vs. Al, showing a current density of ~0.6 A / cm^2. Supplementary Fig. 4e shows XRD patterns of the anode and anode precipitate, indicating that the anode is UAl_3, while the precipitate is a mixture of UCl_3 and UAl_3. The presence of UAl_3 in the precipitate is attributed to the flaking of alloy particles, which suggests that the anode alloy is not mechanically stable enough. Supplementary Fig. 4f shows the XRD patterns of the U-Al alloy anode after re-melting, indicating that the composition of the alloy is UAl_3 and Al. Meanwhile, it can be seen that U-Al alloy is melted together from the insets of Supplementary Fig. 4f, showing that it does require high temperature processing.

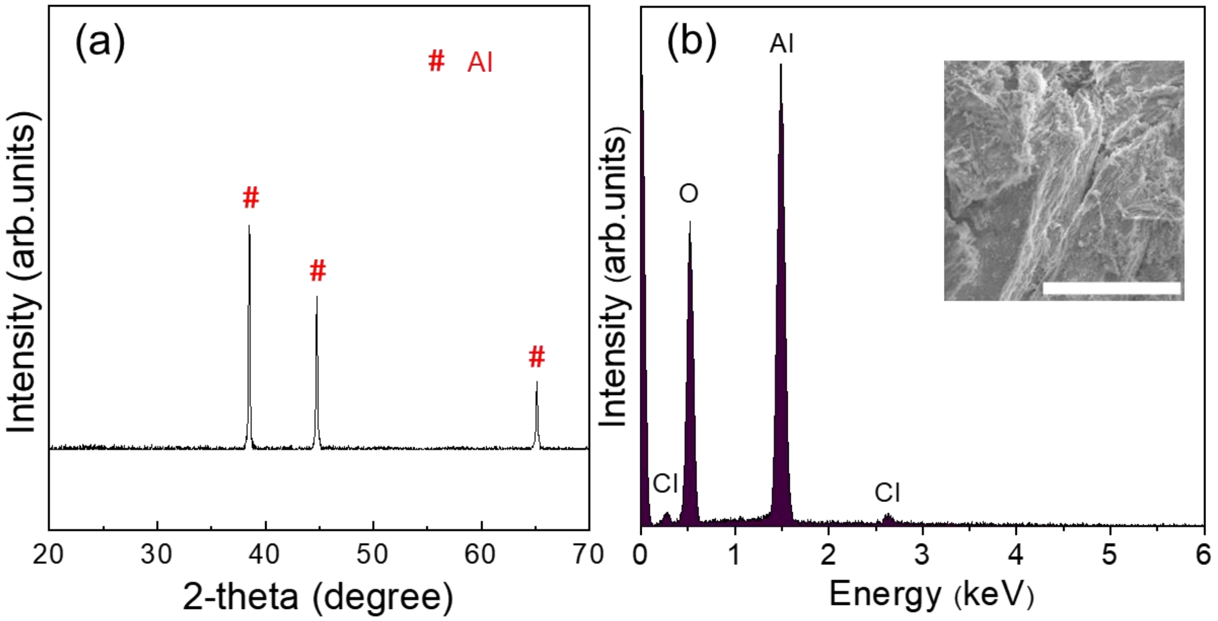

Supplementary Figure 5. Analysis of the cathode product. (a) XRD pattern of the cathode product after water washing and (b) typical EDS analyses; insets are the corresponding SEM image. Scale bar = 100 μm.

In addition to the direct analysis of the cathode after electrolysis, the cathode after water washing is also analyzed, and the results are summarized in Supplementary Fig. 5. The

XRD pattern shows a single pure aluminum phase, as shown in Supplementary Fig. 5a, which is in good agreement with the EDS result (Supplementary Fig. 5b). The cathode has a layered structure according to the SEM image shown in the inset of Supplementary Fig. 5b.

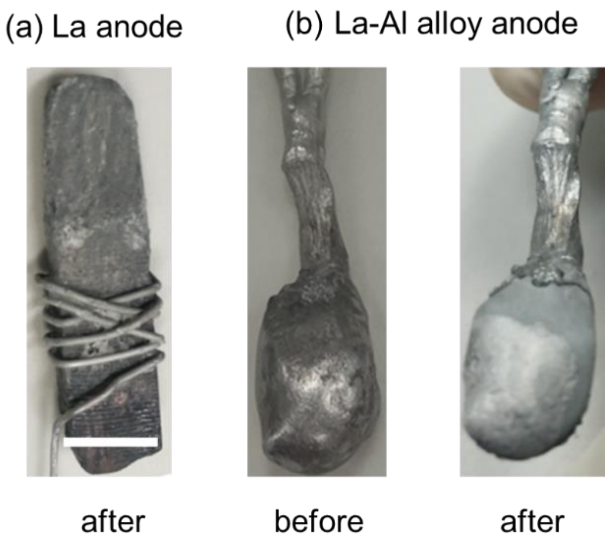

Supplementary Figure 6. Direct observation of the anodes. Photos of (a) the La anode after electrolysis; and the La-Al alloy anode (b) before and after electrolysis. Scale bar = 1 cm.

The photos of the La and La-Al anodes in Supplementary Fig. 6 reveal electrode surface removal during electrolysis.

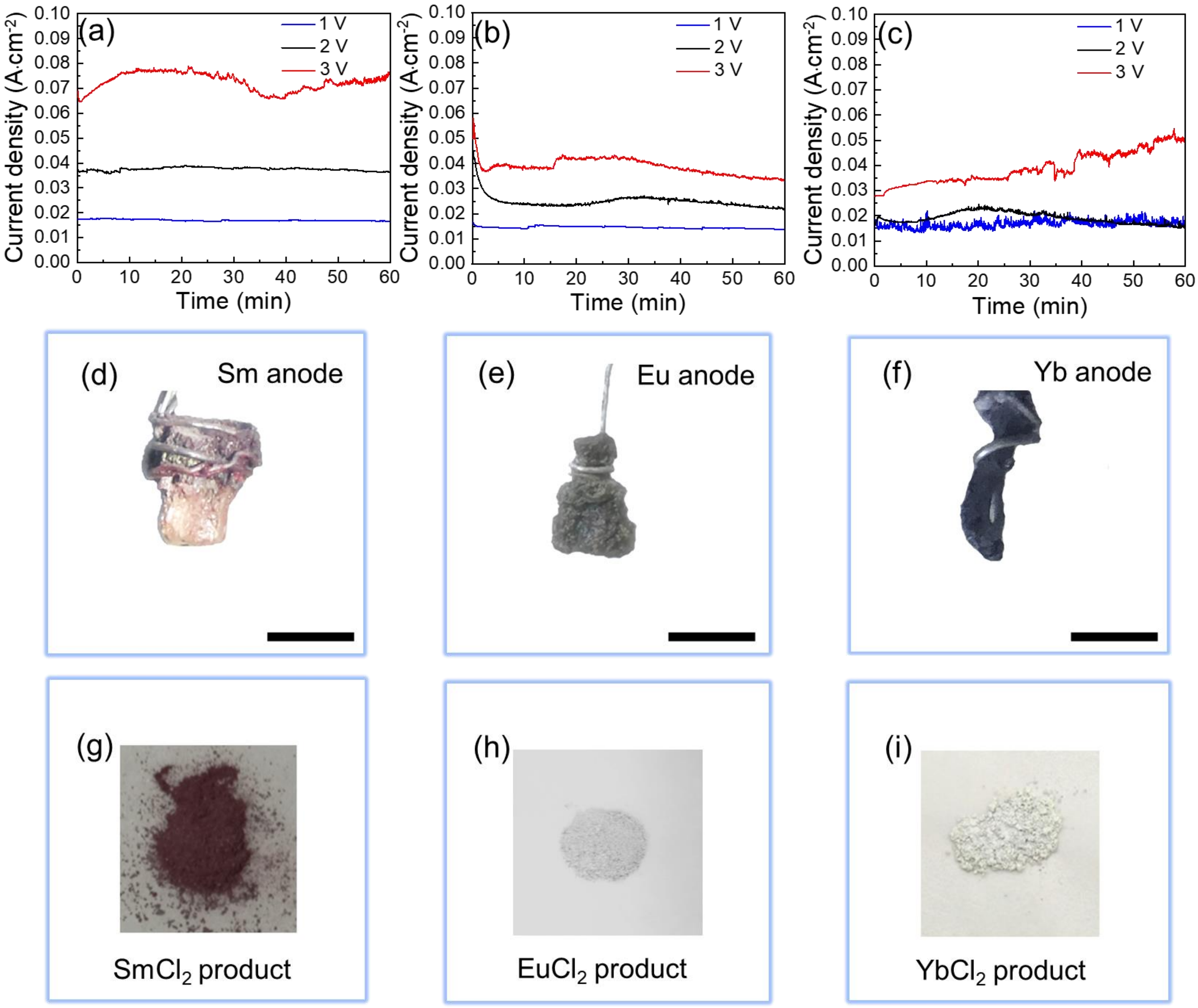

Supplementary Figure 7. IAP process of Sm, Eu and Yb. Current versus time plots in $NaAlCl_4$ molten salt electrolyte at 453 K for anodes (a) Sm, (b) Eu, and (c) Yb. Corresponding photos of the anodes are in (d)-(f), and of the anode precipitates are in (g)-(i). Scale bar = 1cm.

From the electrolysis results for Sm, Eu and Yb anodes shown in Supplementary Fig. 7a-c, it is apparent that for all three metals the current density increases substantially with electrode potential, confirming kinetics highly dependent on anode polarization. Exposure of new anode surfaces during electrolysis is evident in Supplementary Fig. 7d-f, and the uniform color anodic precipitates are shown in Supplementary Fig. 7g-i.

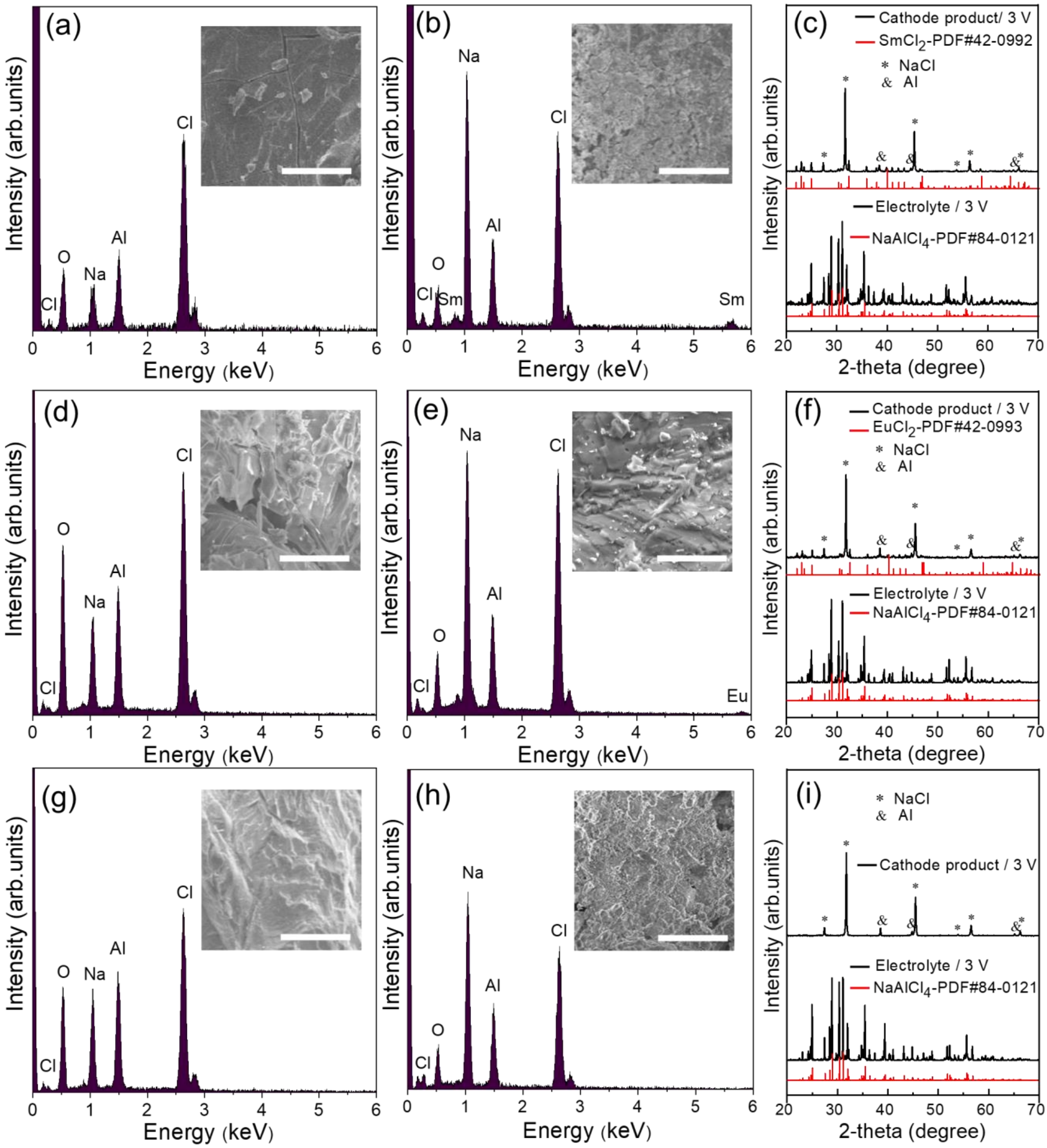

Supplementary Figure 8. Analyses of electrolyte and cathode after IAP. EDS analysis of $NaAlCl_4$ molten salt electrolyte after electrolyzed with anodes (a) Sm, (d) Eu, and (g) Yb at 3 V. EDS analysis of the corresponding cathode products (b), (e), (h); insets are the corresponding SEM images. The corresponding XRD patterns of the electrolyte and the cathode products after electrolysis (c), (f), (i). Scale bar = 100 μm.

In order to explore the cause of recovery loss, the electrolyte after electrolysis with metal anodes Sm, Eu and Yb were analyzed and the results were summarized in Supplementary Fig. 8. It can be seen in Supplementary Fig. 8a that no obvious indication of Sm is observed in $NaAlCl_4$ molten salt electrolyte when Sm anode is used for electrolysis; Similarly, no characteristic peaks due to Eu (Supplementary Fig. 8d) and Yb (Supplementary Fig. 8g) are observed in $NaAlCl_4$ molten salt electrolyte when Eu and Yb anodes are used for electrolysis, indicating the poor solubility of the product in electrolyte, which is consistent with the XRD results (Supplementary Fig. 8c, 8f, 8i).

The cathode product was also analyzed by SEM-EDS and XRD. It is apparent that $SmCl_2$ precipitation is adsorbed on the cathode (as shown in Supplementary Fig. 8b, 8c), resulting in the loss of recovery rate. Similar phenomenon is also observed when Eu anode is used (Supplementary Fig. 8e, 8f). However, no obvious indication of Yb is observed either in electrolyte or cathode (Supplementary Fig. 8h, 8i), so the recovery of Eu is higher than that of Sm and Yb.

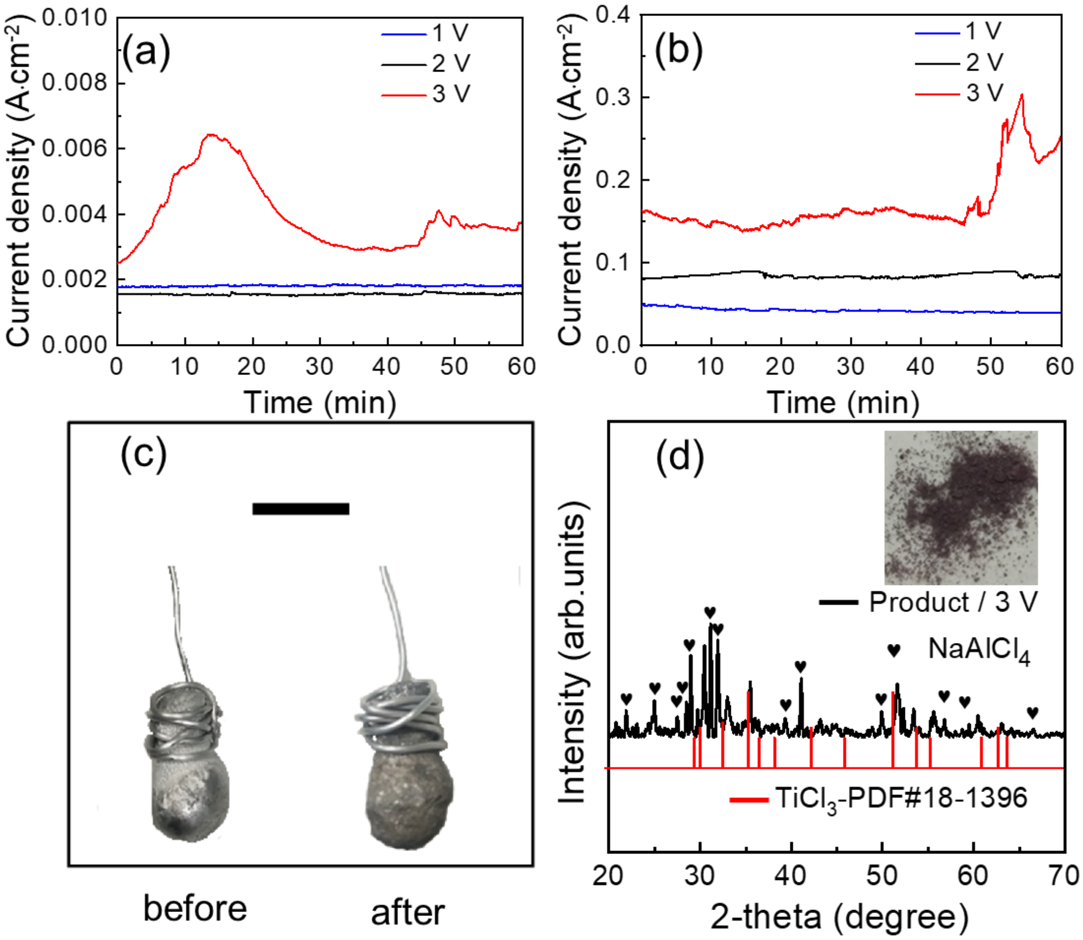

Supplementary Figure 9. IAP process of Ti and Ti-Al alloy. Current versus time plots for (a) Ti and (b) Ti-Al alloy anodes in $NaAlCl_4$ molten salt electrolyte at 453 K; (c) Photos of the Ti-Al alloy anodes before and after electrolysis. Scale bar = 1cm. (d) XRD results for the anode product after electrolysis at 3 V; the inset: corresponding photo of the anode precipitate.

Current versus time curves for Ti and Ti-Al alloy anodes are in Supplementary Fig. 9a and 9b. Only a very low current was observed for the Ti anode at the highest electrode potential of 3 V vs. Al. As for U and La, addition of Al to the Ti anode greatly enhanced the electrolysis rate, with the anode rough and dull after electrolysis as shown Supplementary Fig. 9c. Consistent with the reference results[8], the dark purple anode precipitate is identified as $TiCl_3$ from XRD results in Supplementary Fig. 9d.

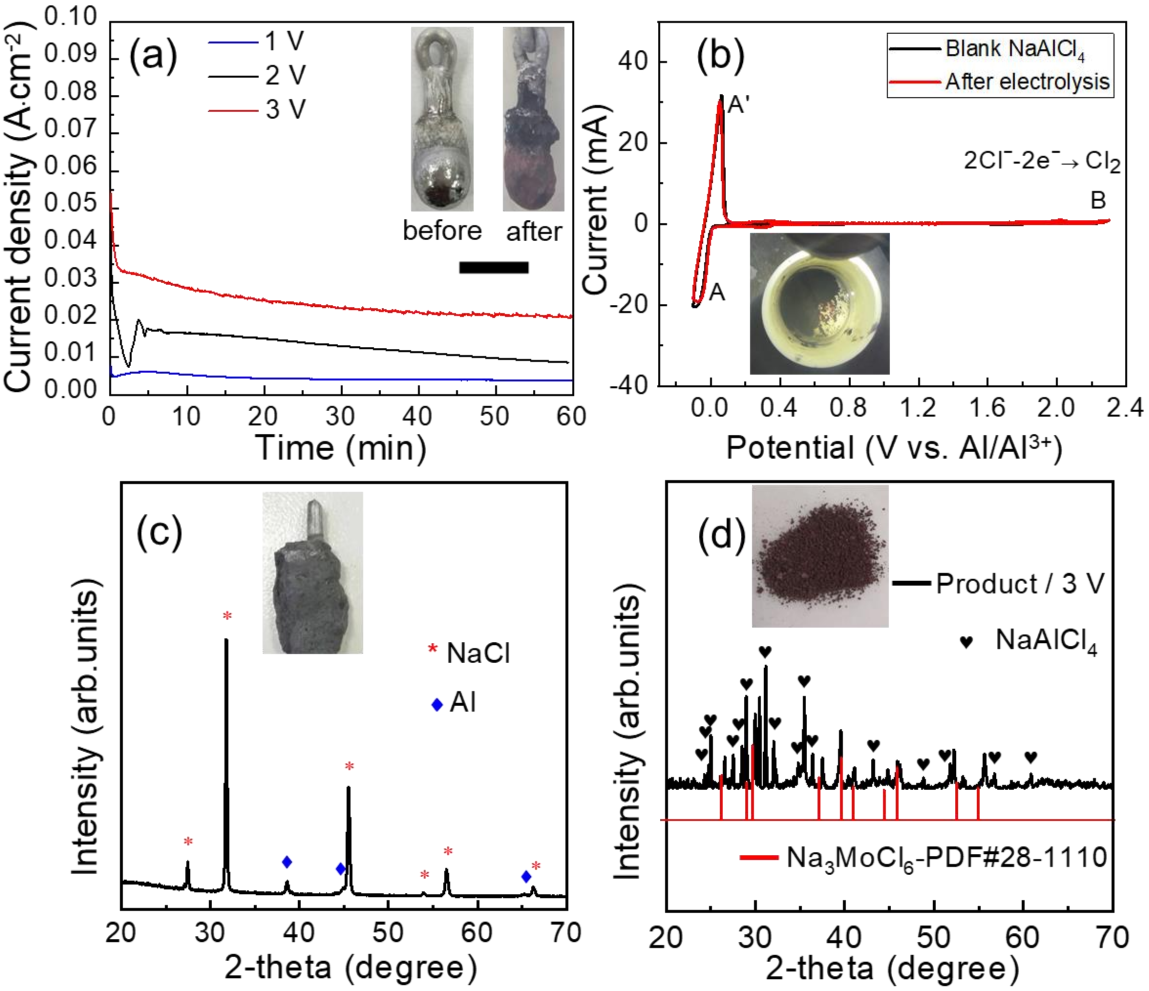

Supplementary Figure 10. IAP process of Mo-Al alloy. (a) Current versus time plots for electrolysis of Mo-Al alloy anode at 1 V, 2 V and 3 V vs. Al in $NaAlCl_4$ molten salt electrolyte at 453 K; insets are images of the anodes before and after electrolysis, respectively. Scale bar = 1cm. (b) CV curves of $NaAlCl_4$ molten salt electrolyte before and after electrolysis; insets are photos looking down into the anode crucible after electrolysis. (c) XRD pattern of the cathode product after electrolysis, with an image of the cathode. (d) XRD results for the anode product after electrolysis at 3 V; the inset: corresponding image of the anode precipitate.

Supplementary Fig. 10a shows current versus time curves for Mo-Al alloy anodes at three potentials, and the current density increases as the electrode potential is increased to 2V and then 3V vs. Al, meanwhile, the photos of the Mo-Al anodes (inset) reveal electrode surface removal during electrolysis. The anode precipitate and electrolyte supernatant after electrolysis are shown in the inset of Supplementary Fig. 10b; the precipitate is easily

separated from the electrolyte by decanting. The CV curves of $NaAlCl_4$ molten salt electrolyte before and after electrolysis are essentially coincident, as in Supplementary Fig. 10b, indicating no obvious electrochemical signal of molybdenum is detected in the CV potential window. Consistent with the CV results, the solubility of molybdenum in the supernatant is small ($6.67*10^{-4}$ mol/ L) analyzed by ICP-OES. Further, no signal of molybdenum is found in the XRD analysis of the cathode (Supplementary Fig. 10c), suggesting that molybdenum does not deposit on the cathode. And the dark red anode precipitate is identified as Na_3MoCl_6 from XRD results in Supplementary Fig. 10d.

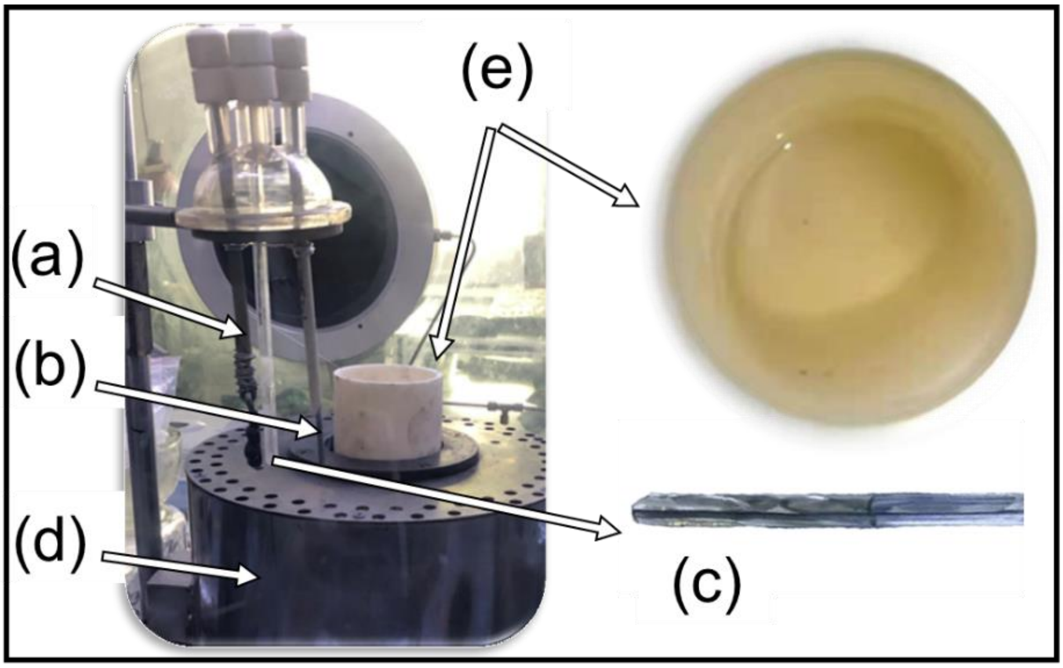

Supplementary Figure 11. The photograph of experimental setup; (a) working electrode, (b) counter electrode, (c) Al reference electrode, (d) electric furnace, (e) corundum crucible with electrolyte.

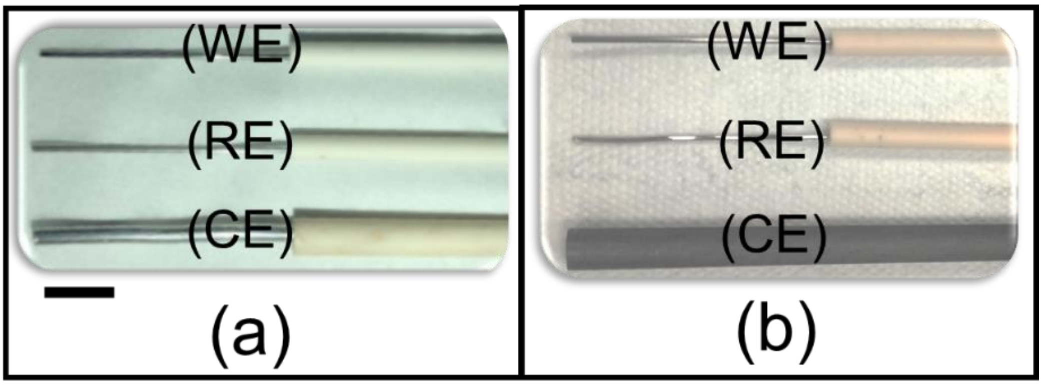

Supplementary Figure 12. The three electrodes system for CV tests. (a) Electrodes for CV tests in $NaAlCl_4$ molten salt electrolyte at 453 K. (b) Electrodes for CV tests in KCl-LiCl molten salt electrolyte at 723 K. Scale bar = 1cm.

Supplementary Tables

Supplementary Table 1. Fitting Parameters from Least-Squares Fitting Analysis of UCl_3 EXAFS Spectra in Supplementary Figure 3.

Sample	Shell	CN[a]	R(Å)[b]	σ^2/(Å^2)[c]	E/(eV)[d]	R-factor[e]
UCl_3	U-Cl	6	2.92	0.010	5.5	0.018
	U-Cl-Cl-U	36	4.63	0.003	5.5	

[a] Coordination numbers. [b] Radial distance. [c] Debye−Waller factor. [d] Energy shift relative to the calculated Fermi level. [e] Goodness-of-fit indicator.

Supplementary References

1. L. G. Boxall, H. L. J., and R. A. Osteryoung. Solvent Equilibria of AlCl -NaCl Melts. *J. Electrochem. Soc*. **120**, 233-231 (1973).
2. Zachariasen, W. H. The UCl_3 Type of Crystal Structure. *J. Chem. Phys*. **16**, 254-254 (1948).
3. Liu, K., Ma, Y., Kang, M., and Wang, B. Facile visualization of the initial nucleation and growth of an active metal electrodeposited in a high temperature molten salt using a detachable disk electrode. *Electrochem. Commun*. **117**, (2020).
4. Till, C. E., and Chang, Y. I. *Plentiful Energy: the Story of the Integral Fast Reactor* (CreateSpace).(2011).
5. Hoover, R. O., Shaltry, M. R., Martin, S., Sridharan, K., and Phongikaroon, S. Electrochemical studies and analysis of 1–10wt% UCl_3 concentrations in molten LiCl–KCl eutectic. *J. Nucl. Mater*. **452**, 389-396 (2014).
6. Liu, Y.-L., Yuan, L.-Y., Zheng, L.-R., Wang, L., Yao, B.-L., Chai, Z.-F., and Shi, W.-Q. Confirmation and elimination of cyclic electrolysis of uranium ions in molten salts. *Electrochem. Commun*. **103**, 55-60 (2019).
7. Liu, Y.-L., Ye, G.-A., Yuan, L.-Y., Liu, K., Feng, Y.-X., Li, Z.-J., Chai, Z.-F., and Shi, W.-Q. Electroseparation of thorium from ThO_2 and La_2O_3 by forming Th-Al alloys in LiCl-KCl eutectic. *Electrochim. Acta*. **158**, 277-286 (2015).
8. Sekimoto, H., Nose, Y., Uda, T., and Sugimura, H. Preparation and properties of trivalent titanium compounds, $TiCl_3$ and TiOCl. *High Temp. Mat. Pr-isr*. **30**, 435-440 (2011).
